# Mapping the wildland-urban interface in California using remote sensing data

**DOI:** 10.1038/s41598-022-09707-7

**Published:** 2022-04-06

**Authors:** Shu Li, Vu Dao, Mukesh Kumar, Phu Nguyen, Tirtha Banerjee

**Affiliations:** Department of Civil and Environmental Engineering, University of California, Irvine, Irvine, CA 92697 USA

**Keywords:** Natural hazards, Environmental social sciences

## Abstract

Due to the mixed distribution of buildings and vegetation, wildland-urban interface (WUI) areas are characterized by complex fuel distributions and geographical environments. The behavior of wildfires occurring in the WUI often leads to severe hazards and significant damage to man-made structures. Therefore, WUI areas warrant more attention during the wildfire season. Due to the ever-changing dynamic nature of California’s population and housing, the update frequency and resolution of WUI maps that are currently used can no longer meet the needs and challenges of wildfire management and resource allocation for suppression and mitigation efforts. Recent developments in remote sensing technology and data analysis algorithms pose new opportunities for improving WUI mapping methods. WUI areas in California were directly mapped using building footprints extracted from remote sensing data by Microsoft along with the fuel vegetation cover from the LANDFIRE dataset in this study. To accommodate the new type of datasets, we developed a threshold criteria for mapping WUI based on statistical analysis, as opposed to using more ad-hoc criteria as used in previous mapping approaches. This method removes the reliance on census data in WUI mapping, and does not require the calculation of housing density. Moreover, this approach designates the adjacent areas of each building with large and dense parcels of vegetation as WUI, which can not only refine the scope and resolution of the WUI areas to individual buildings, but also avoids zoning issues and uncertainties in housing density calculation. Besides, the new method has the capability of updating the WUI map in real-time according to the operational needs. Therefore, this method is suitable for local governments to map local WUI areas, as well as formulating detailed wildfire emergency plans, evacuation routes, and management measures.

## Introduction

The process of suburbanization in the United States, which has continued since World War II, has dramatically increased the impact of human activities on natural ecosystems^1,2^. The urban sprawl caused by the migration of population to the suburbs has intensified the ingression of human structures into wildlands, forests, and habitats^3^. To monitor and evaluate the impact of these human activities on local climate and environment, the area where human structures and wildland vegetation coexist either adjacent or interspersed with each other were defined as Wildland-Urban Interface (WUI)^4,5^. In California, one of the most notable features of WUI is that they are perceived as high-risk areas of human-caused wildfires due to the accumulation of wildland vegetation, the concentration of flammable human structures, and the strewing of sparks left by human activities^6^. Although most wildfires occur in uninhabited wildland, wildfires ignited or spread into WUI areas pose a more significant threat to human lives and assets due to the proximity of human community and wildland fuels^7–9^. Besides, the disturbance to local species and the introduction of invasive species caused by the construction and development of human communities weaken local ecosystems’ resistance and resilience to wildfires^10^. Therefore, WUI area is of great concern in wildfire prevention and management.

The distribution of WUI in the United States is widespread and continues to rise. From 1990 to 2010, WUI area in the contiguous United States increased rapidly from 7.2 to 9.5$\%$, which caused a 9.6$\%$ increase in the number of houses and a 8.5$\%$ increase in land area within in the new WUI, accompanied by the increase in housing and population^11^. During the same time span, the WUI area in California increased from 26,263 $\mathrm {km^{2}}$ to 27,255 $\mathrm {km^{2}}$, with an increase of about 3.8$\%$. By 2010, the number of houses within the WUI had grown to 4.46 million, and the population had grown to 11.2 million in California, making it the state with the largest number of houses and population in the WUI^12^. The growth of WUI not only increased the risk of fire ignitions but also increased the difficulty of fire extinction, because in these areas, firefighters’ top priority is to protect people and assets^13^. Given the above, accurately mapping and timely updating the WUI region to provide firefighters and emergency responders with more complete and practical WUI maps is of prime importance^14^.

Based on the definition of WUI from the Federal Register^15^, and the current common operational mapping method^16,17^, areas with housing density greater than 6.17 houses/$\mathrm {km^{2}}$ and vegetation cover greater than $50\%$ are classified as WUI. The housing density is calculated using housing counts in Census blocks, and the threshold is 1 house/40 acre (6.17 houses/$\mathrm {km^{2}}$). The vegetation proportion is calculated by extracting vegetation area from the National Land Cover Database (NLCD) within each Census block, the threshold of vegetation is $>50\%$ for intermix and $>75\%$ for interface^15^. It means that the WUI area should meet requirements of *high-density houses surrounded by high-density wildland vegetation* (50$\%$ of vegetation area in the Census block) or *high-density houses adjacent to, that is within 1.5 miles (2.4 km) of a large tract of contiguous wildland vegetation* (75$\%$ of vegetation area in the Census block).

The current common WUI mapping methods heavily rely on the threshold of building density and vegetation proportion. However, because of the limitations in the update-frequency (10 years for Census data) and the precision of housing and vegetation data, there are uncertainties in the current data sources and mapping methods, especially in calculating housing density^17^. Radeloff et al.^5^ mapped WUI areas of all states in the contiguous United States based on housing statistics provided by the Census data in 2010 and vegetation cover provided by the NLCD. The minimum unit of housing density in this zonal mapping method was Census block, and the zone modifiable areal unit problem cannot be avoided^17^. Meanwhile, this method removes all the area of public land to get a more precise housing density. However, there are also human structures on public lands, such as power-lines and airports, and their effect is completely ignored. Subsequently, as an improvement to the zonal mapping method, Platt^18^ used points mapped from the parcel centroids representing building locations to calculate housing density in WUI mapping. The limitation of this method is that these points were not the actual locations of the buildings and would still be erroneous. Bar-Massada et al.^17^ mapped WUI directly from the buildings’ location and calculated housing density by “circular moving window analysis”. It divides the studied region by grids first and then overlaying a circular moving window to calculate the mean housing density for all the grids in the window. The mean housing density would be assigned to the central grid. Although WUI maps can be customized based on needs, defining appropriate window sizes in different spatial scale is a challenge. Besides, traditional housing density data are updated at long intervals, as Census data are collected and updated every ten years. The current update frequency of WUI maps is far behind its growth rate.

Efforts to digitize spatial housing locations and use them for WUI mapping or wildfire risk assessment include extracting building locations from GPS records^19^, and mapping structures from high-resolution digital orthophotographs^20^. Moreover, with the improvement of the quality of remote sensing data in terms of acquisition efficiency and resolution, it has become possible to extract detailed housing and vegetation boundaries from such datasets. For example, Caggiano et al.^21^ explored the feasibility of detecting individual buildings from National Aerial Image Program imagery using Object Based Image Analysis; Johnston and Flannigan^22^ used structure locations from CanVec+, which were derived from a variety of remote sensing products, and wildland fuels from Land Cover 2000 dataset, which was based on Landsat 5 and Landsat 7 iamges, to map the interface areas in Canada; Alcasena et al.^23^ used remote sensing data provided by Spanish national topographic platform (BTN25) map to identify the housing locations and the vegetaion map from land parcel identification system to identify forest land polygons.

Over the past few years, Microsoft has made significant efforts in applying deep learning, computer vision and AI to mapping and leveraging the power of machine learning in analyzing satellite imagery to trace the shape of buildings across the country. The building footprint dataset released by Microsoft in 2018 contains 129,591,852 buildings, covering the entire United States; and it is available to download free of charge. It used Deep Neural Network and residual neural network (ResNet34) with segmentation techniques (RefineNet up-sampling) to detect individual building footprints from their imagery data^24^. In terms of the vegetation data, the LANDFIRE program from United States Geological Survey (USGS) utilizes Landsat 7 Enhanced Thematic Mapper Plus and Landsat 8 Operational Land Imager products to provide national scale vegetation, fuel, and fire regime data^25^. The fuel vegetation cover data from the LANDFIRE database not only update more frequently than NLCD maps, but also directly provide pixel-level vegetation cover percentage, simplifying and reducing the computational cost in WUI mapping.

Using the above-mentioned data, the WUI map in California as well as the WUI mapping method can be updated. Our goal in this paper is to (1) use housing footprints from Microsoft and fuel vegetation data from the LANDFIRE program, combining the definition of WUI and, based on the characteristics of the data, design a practical WUI rendering method; (2) compare the new WUI mapping method against previous methods, and analyze if there are improvements to the previous maps; (3) combining with the historical wildfire record, analyze the usefulness of the new WUI map in wildfire risk assessment.

## Results and discussion

### Threshold determination

**Figure 1:**
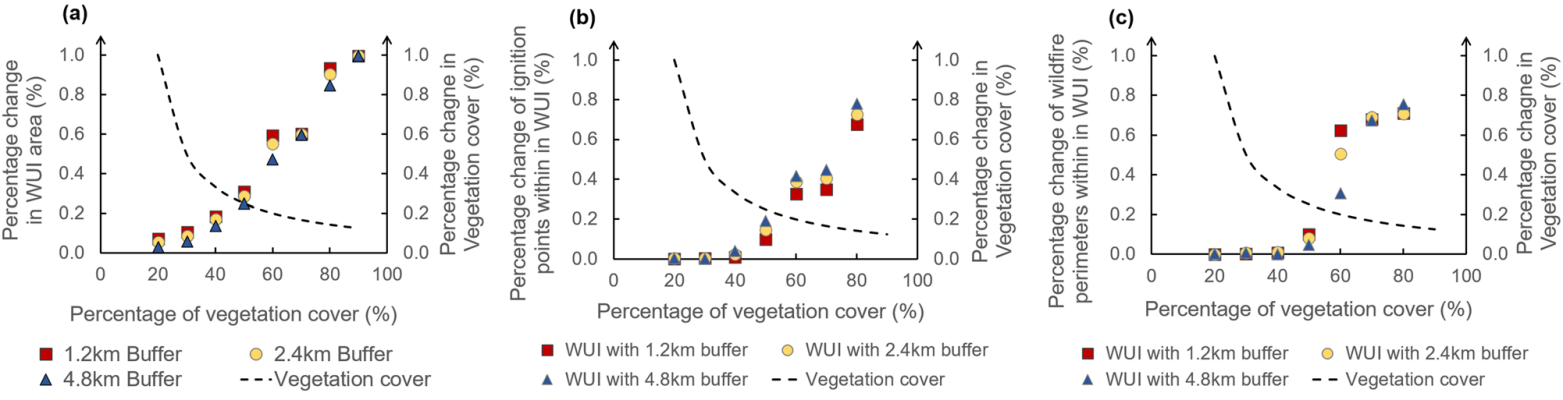
Percentage change of WUI area and wildfires with vegetation cover: (**a**) WUI area; (**b**) ignition points within WUI; (**c**) burned areas within WUI. The x axes represent the percentage of vegetation cover included in the WUI mapping. The colored dots represent the WUI-RS area, ignition points and wildfire perimeters included in the WUI-RS under different vegetation cover and buffer radius. To test the sensitivity of the WUI map, the vegetation cover changed by $10\%$ at a time, and its rate of change is on the secondary Y axis and represented by dash lines.

The threshold of the two main components in the WUI definition—vegetation cover and the distance between housing and high-density vegetation, were tested using the one factor at a time (OFAT) approach^16^. The validity of the map was evaluated by three indicators: the percentage change of WUI area with changes in the parameter value, wildfire ignition points within WUI and wildfire perimeters within WUI. When the percentage changes of the indicators are smaller than the percentage change of the WUI components, the threshold can be seen as stable^5^. The results are shown in Fig. 1. For the vegetation cover, when the threshold is lower than $40\%$, the change in the new WUI area is relatively stable; on the other hand, when the threshold is lower than $50\%$, the change in the ignition points and the perimeters are relatively stable. Thus $40\%$ of vegetation cover was selected as the threshold. More specifically, the housing within or next to the wildland area with vegetation coverage higher than $40\%$ were seen as WUI area. In terms of how close the housing should be to the wildland vegetation, results show that the change of $50\%$ increase or decrease based on 2.4 km does not have a drastic effect on the three validity indicators, and the range of percentage change in each indicator is within $50\%$. Therefore, 2.4 km is reasonable both conceptually and operationally in this method. Since there is no restriction on the density of houses in this method, the area of buffer directly determines whether the houses are included in WUI. Overall, WUI area in this new operational method (WUI-Remote Sensing, abbreviated as WUI-RS) was defined as the area in which man made structures are surrounded by or within 2.4 km of wildlands where the vegetation cover is higher than $40\%$.

### Latest WUI maps

Based on the above optimal thresholds, we mapped the WUI area in California using Microsoft housing footprint data in 2018 and LANDFIRE fuel vegetation cover in 2018, which we designate as remote sensing based WUI (WUI-RS). Since the WUI map generated with remote sensing data can be updated at any time as long as we delineate the housing footprints from the satellite images, we did not label the year the map was drawn.

There are currently two common WUI maps used in California: one was released by Martinuzzi et al.^12^ in 2015 using Census data in 2010 NLCD data in 2006, hereinafter WUI-USFS (2010); the other was released by the State of California and the Department of Forestry and Fire Protection (CAL FIRE) using several internal data sources for the 2015 Assessment of Forest and Rangelands, hereinafter WUI-FRAP (2010). To compare our mapping approach with the existing mapping approaches we have to overcome one obstacle which is that the WUI-USFS is from 2010 as they used the 2010 Census data. In order to facilitate a fair comparison, we updated the WUI-USFS map to 2020 as well. We followed the common zonal-based mapping method^12,16^ and used the latest Census data in 2020 to map the WUI area in California as well and designate it WUI-USFS (2020).

**Figure 2:**
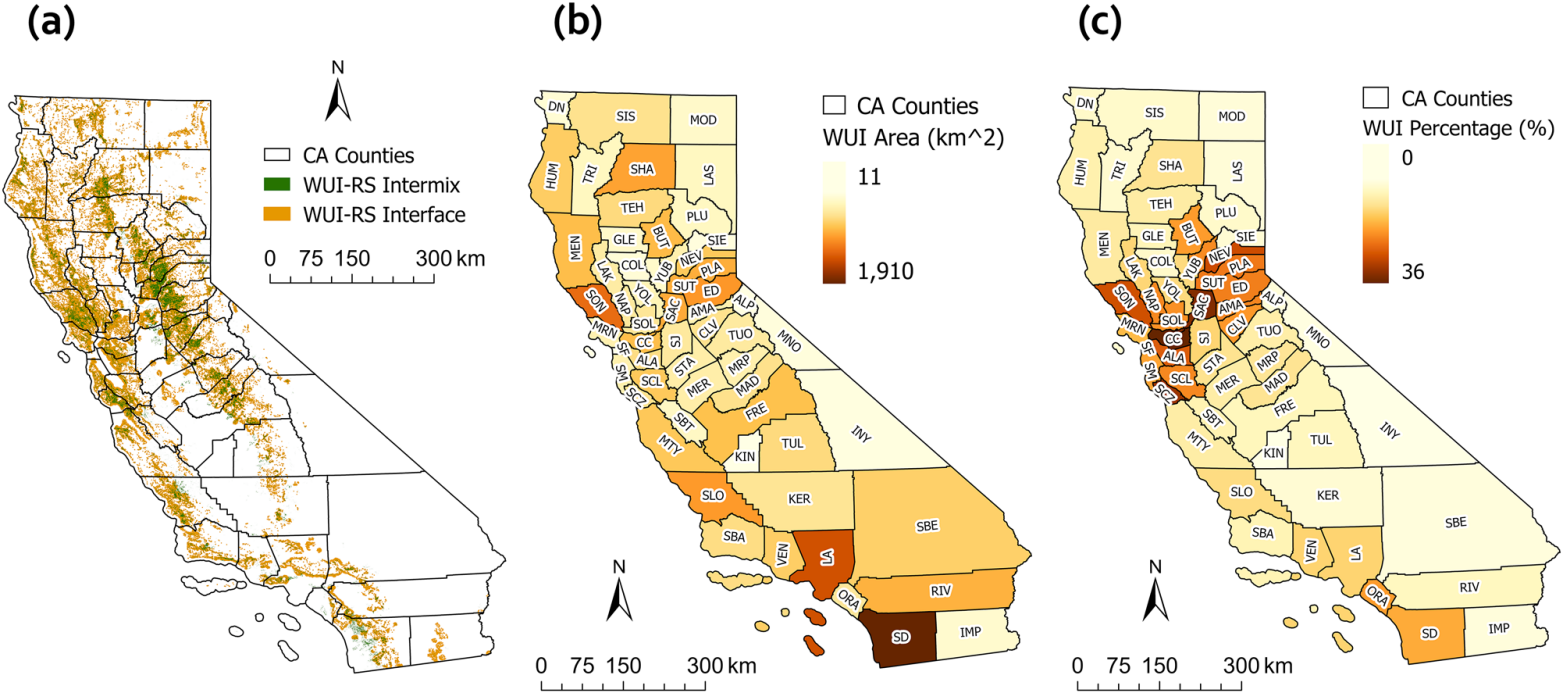
WUI-RS map in 2018 and the spatial distribution of WUI-RS area in California: (**a**) WUI-RS map in 2018; (**b**) WUI-RS area in each county; (**c**) percentage of WUI-RS cover in each county.

The map of the WUI-RS is shown in Fig. 2a. The WUI-RS map covers an area of 28,575 $\mathrm {km^{2}}$ in California, with 55.21$\%$ intermix area (15,776 $\mathrm {km^{2}}$) and 44.79$\%$ interface area (12,799 $\mathrm {km^{2}}$), accounting for 6.74$\%$ of the land area in CA (423,971 $\mathrm {km^{2}}$). About five million housing units, which accounts for 45.13$\%$ of total housing in California are included in the WUI-RS. The distribution of the new WUI is concentrated along the western coastline and to the west of the Sierra Nevada Mountain range. It is sparse in the central and southeastern California, because most of the San Joaquin Valley in the Central California have been developed and planted, and Southeastern California has vast tracts of barren land and very few human structures.

At the county level, Fig. 2b,c shows the area and percentage of WUI in each county. The San Diego (SD), Los Angeles (LA) and Sonoma (SON) counties contains largest WUI area which are 1909 $\mathrm {km^{2}}$, 1400 $\mathrm {km^{2}}$, 1225 $\mathrm {km^{2}}$ separately. The Contra Costa (CC), Sacramento (SAC) and Santa Cruz (SCZ) counties in the northern California have the highest percent of WUI which accounts for 36.18$\%$, 33.64$\%$ and 31.44$\%$ of their land area separately.

**Figure 3:**
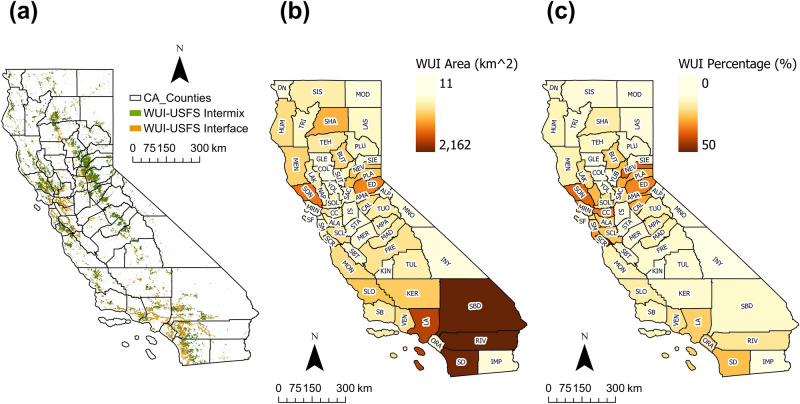
WUI-USFS map in 2020 and the spatial distribution of WUI-USFS area in California: (**a**) WUI-USFS map in 2020; (**b**) WUI-USFS area in each county; (**c**) percentage of WUI-USFS cover in each county.

The map of the WUI-USFS(2020) is shown in Fig. 3a. The total area of the updated WUI-USFS map is about 29,343 $\mathrm {km^{2}}$, including 70.22$\%$ intermix area (20,605 $\mathrm {km^{2}}$) and 29.78$\%$ interface area (8738 $\mathrm {km^{2}}$). It shows the similar spatial patterns with the WUI-RS map, which are along the western coast and to the west of the Sierra Nevada mountain, while the density of the WUI area is much lower than WUI-RS. The reason for this difference is that the new method put more emphasis on the presence of man made-structures rather than structure density. Thus the WUI-RS map include the area with high density vegetation and low density human-structures as well. Compared to the WUI map of California in 2010 released by USFS, the total area of WUI in 2020 increased by approximately 2000 $\mathrm {km^{2}}$, with the similar spatial distribution. The growth in WUI area over the decade was mainly driven by an increase in the number of houses in California.

Figure 3b,c show the statistics of updated zonal-based WUI area and their percentage of land area in each county. Three counties in sourthern California: the San Bernardino (SBD), Riverside (RIV) and San Diego (SD) county have the largest WUI area, especially compared to the WUI-RS map. However after dividing the total land area in each county, the spatial distribution of the percentage of the WUI areas, which is shown in Fig. 3c, has similar patterns with WUI-RS. The counties in the central California and on both side of the Sierra Nevada Mountain have high percentage of WUI area.

**Figure 4:**
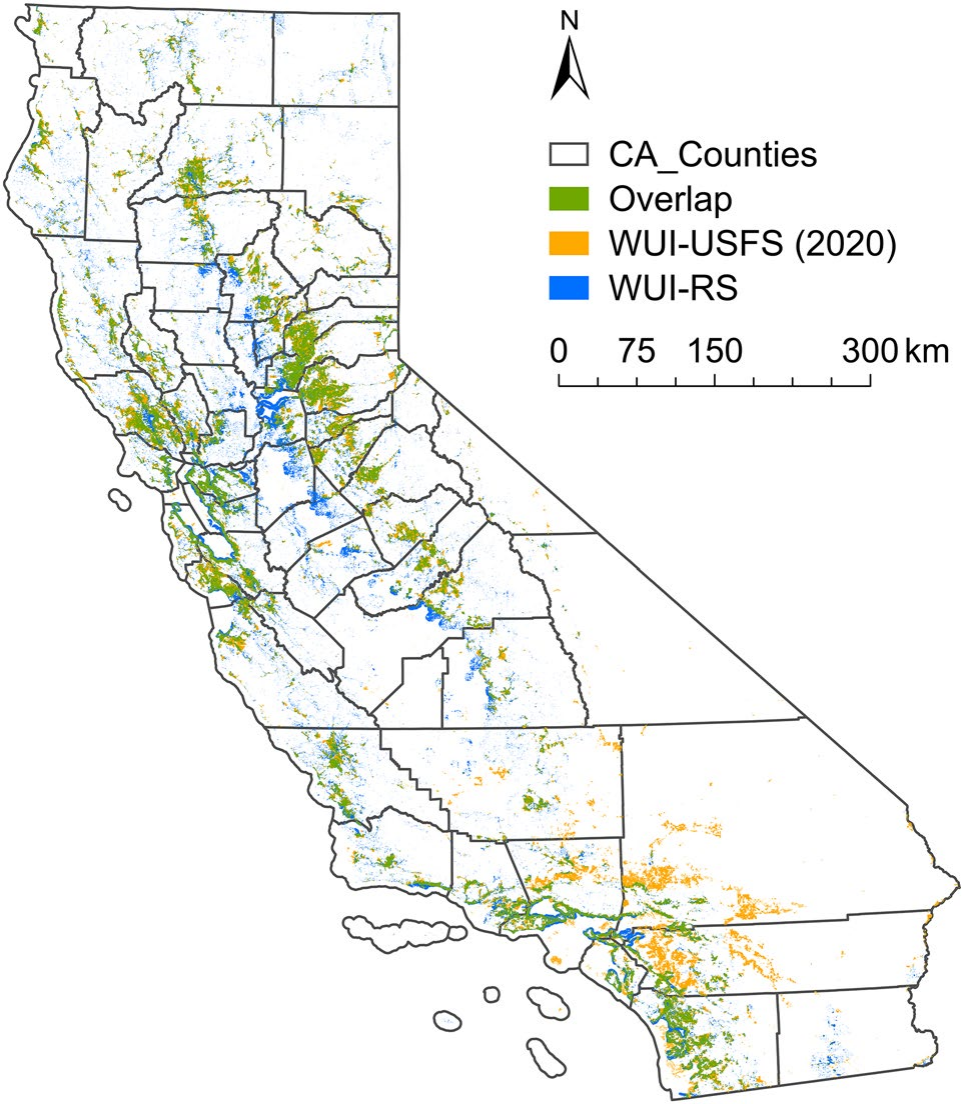
Overlaps and differences between WUI-RS and WUI-USFS (2020).

The overlaps between the most recent WUI-RS map and WUI-USFS map is shown in Fig. 4. The total area of the overlap is 22,245 $\mathrm {km^{2}}$, which accounts for 77.85 $\%$ of the total area of WUI-RS and 75.81 $\%$ of the total area of WUI-USFS (2020). The blue areas in Fig. 4 are the unique part in the WUI-RS map, which are concentrated in central California, west of the Sierra Nevada Mountains. They are not included in the WUI-USFS map because of the low housing density. The orange areas in Fig. 4 show the unique part in the WUI-USFS map, which are concentrated in southern California. These areas did not meet the vegetation density threshold of the WUI-RS map. In general, the high percentage of overlaps between these two maps indicate the validity of the WUI-RS map drawn by using the selected remote sensing data and the tested threshold in this study.

### Comparison between different WUI maps

**Figure 5:**
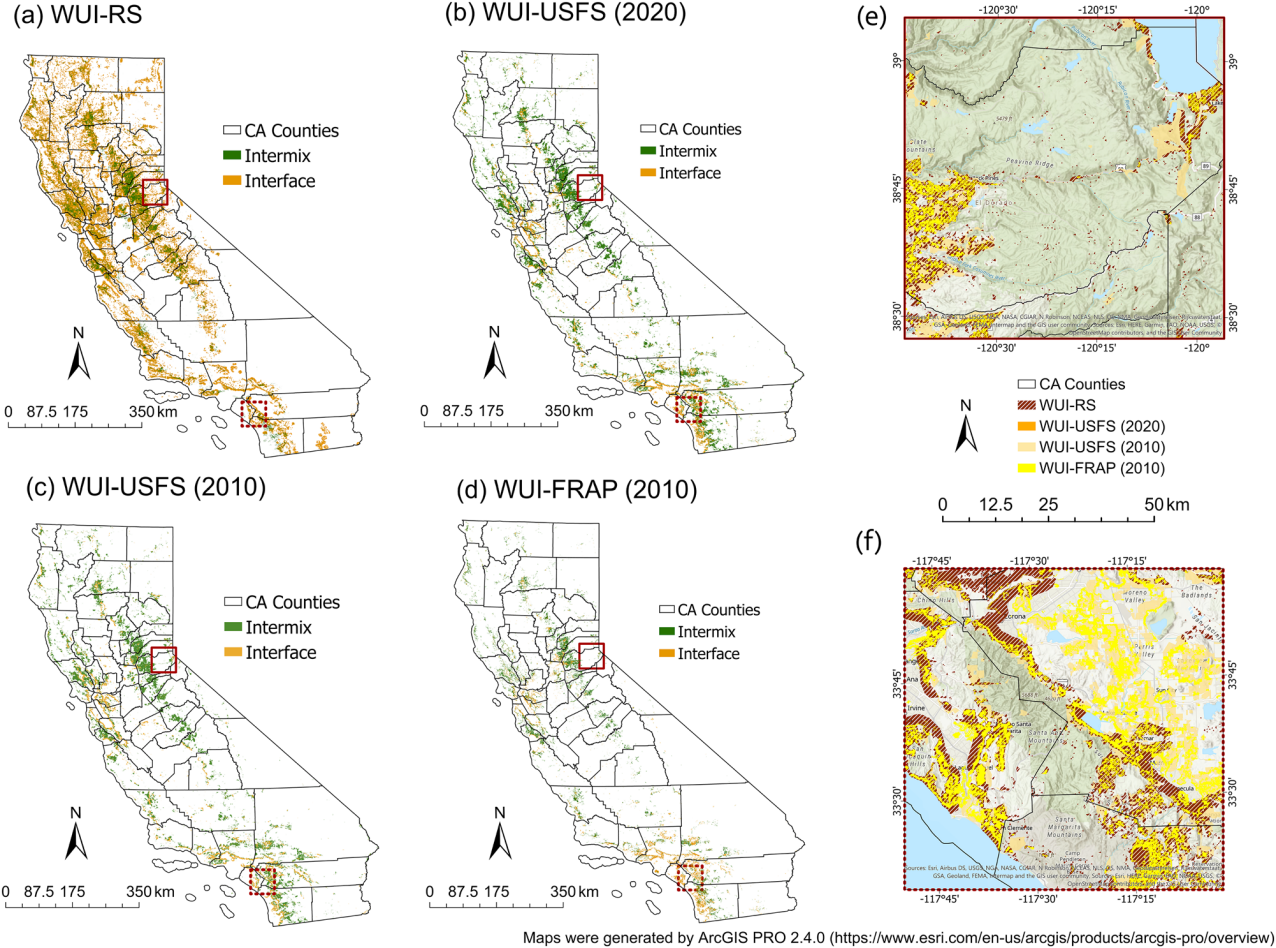
Comparison between (**a**) WUI-RS, (**b**) WUI-USFS (2020), (**c**) WUI-USFS (2010) and (**d**) WUI-FRAP (2010). The detailed comparison among these four maps of two selected enlarged regions are shown in (**e**) and (**f**). Maps were generated by ArcGIS Pro 2.4.0 (https://www.esri.com/en-us/arcgis/products/arcgis-pro/overview).

Different types of WUIs vary widely in their definitions and applications, each of which has its own scope of application. We compared the rendering methods, applications and spatial patterns of WUI-RS, WUI-USFS (2020), WUI-USFS (2010) and WUI-FRAP (2010) in Table 1 and Fig. 5. The comparison here is to demonstrate the differences and analyze the causes. Additionally, the extent and coverage of different WUI maps are dependent to some extent on the criteria used to map them in the first place. However, in our mapping approach the selection of the threshold criteria are not completely arbitrary and based on statistical approaches such as the OFAT technique as discussed earlier.

**Table 1:** Comparison of different WUI mapping methods.

	WUI-RS (Our approach)	WUI-USFS	**WUI-FRAP**
Update frequency	In real time	10 years (limited by Census data)	Only released one map for the 2015 Assessment of Forest and Rangelands
Inputs	Housing footprints (Microsoft); fuel vegetation cover (LANDFIRE)	Housing density (Census housing data); wildland vegetation (NLCD)	Housing density; Fire Hazard Severity Zones; Unimproved Parcels; and Vegetation Cover (all the data are from other FRAP programs)
Operation definition	The area in which structures are surrounded by (interface) or within (intermix) 2.4 km of wildlands where the vegetation cover $\ge$ 40$\%$ (obtained using statistical analysis)	Interface: at least one structure per 40 acre, and located <2.4 km of an area $\ge$ 5 $\mathrm {km^{2}}$ in size that is $\ge$ 75$\%$ vegetated Intermix: at least one structure per 40 acre, and wildland vegetation cover $\ge$ 50$\%$	Interface: at least one structure per 20 acre; in moderate, high, or very high Fire Hazard Severity Zone; Not dominated by wildland vegetation; Spatially contiguous groups of 30m cells that are 10 acres and larger Intermix: not interface; one structure per 20 acres to one structure per 5 acres; at least one structure per 5 acres and dominated by wildland vegetation; In moderate, high or very high Fire Hazard Severity Zone; Improved parcels only; Spatially contiguous groups of 30m cells that are 25 acres and larger
Scope of application	Provide the spatial patterns of WUI accurate to individual houses in California, pinpoint specific WUI areas to show the fire risk and develop targeted management strategies	Provide the extent and locations of WUI areas at the state level, and summarize the statistics at the state to federal level	Provide the overall pattern of WUI development at the county level in California, and compare counties in terms of development patterns
Advantages	(1). The operation definition is straight- forward and simplified, eliminating the calculation of housing density and vegetation cover; (2). WUI-RS can be updated in real- time as needed; (3). WUI-RS is accurate to an individual building and and removes the reliance on ad-hoc thresholding criteria.	(1). The input data were available and consistent nationwide (2). It is feasible to provide the large- scale statistics summaries	(1). Take fire hazards in to account in mapping process (2). Instead of using a single housing density threshold, it refines the housing density into four levels.
Limitations	(1). The vegetation cover data were derived from the LANDFIRE database directly, we did not calibrate it in this research; (2). The new set of thresholds in mapping method only applicable in California, mapping other areas will require area specific calibration.	(1). The maps are not directly comparable with those from earlier decades due to changes in census block boundaries^12^; (2). The shape and area of census blocks are inconsistent, and it will introduce bias^17^; (3). The low density development areas were not factored into the mapping process^18^.	(1). Until the dataset is refined through a field review process, it is not suited for WUI designations for individual houses or neighborhoods; (2). Both the input data and the mapping method are applicable only in California.

In general, all types of WUI areas are concentrated along the Sierra Nevada Mountain and the West Coast. These common features demonstrate that although the new mapping method simplifies the operation steps, it still successfully captures the distribution characteristics of WUI. In Fig. 5e,f, we selected two representative concentrated WUI regions in northern and southern California, presenting two types of general spatial distribution differences among the WUI maps. Figure 5e shows an area in Northern California which has small fragments of WUI-RS but not the other types of WUI. This region belongs to the Eldorado National Forest, dominated by high density of trees. Despite the low housing density, the wildfire ignition points were concentrated here between from 2000 to 2019, and there have been large wildfires in the two decades. The WUI-RS map includes such low-density houses surrounded by high-density vegetation, supplementing these piecemeal WUI areas with potentially high wildfire risk that would have been missed by previous methods. Besides, Fig. 5f shows evident differences in different types of WUI close to the developed areas in Southern California which are dominated by shrubs. In particular, the northeastern region close to the mountains in this figure was treated as non-WUI in the WUI-RS map. This is due to the fact that most of the shrubs cover in this area are under 40$\%$ and are not continuous. At the same time, the vast developed area impede this whole region being classified as high-density wildland vegetation. From the perspective of wildland fire risk, no human-caused wildfires ignited in this area from 2010 to 2019, thus this difference is deemed acceptable.

Compared with WUI mapping methods of WUI-USFS and WUI-FRAP predefining a threshold of housing density, WUI-RS treated each house independently, as the location and the perimeter of houses are known from the building footprint data. The current common WUI mapping methods follow the definition of WUI from the Federal Register, adopting 6.17 houses per $\mathrm {km^{2}}$ as the minimum housing density threshold in WUI, which eliminate the areas with low density buildings adjacent to or within wildland. However, the study by Syphard^26^ demonstrate that the smaller and more isolated housing clusters have higher risk of property loss due to wildfires. Therefore we treated all the buildings within a certain distance of high-density fuel vegetation cover as WUI in this study. This treatment also eliminates the requirement of housing density calculation which is complicated by zoning issues and the uncertainty due the the variation of housing density threshold^16^. Using the fuel vegetation cover (FVC) data from LANDFIRE directly, our new method also gets rid of the vegetation density calculation.

To contextualize the WUI mapping with historical wildfire activity, the number of historical wildfire ignition points and the burned area within WUIs were calculated within ten years of each of the three maps being most effective. As shown in Table 2, WUI-RS captures the majority of human-caused ignition points, and it has the highest percentage to the total ignition points among four WUI maps. In terms of the burned area within WUI, none of the four maps included much of human-caused wildfire area (less than 10$\%$ in general). This is because WUI maps are primarily used to identify houses and human structures with a high risk of igniting wildfires or be affected by wildfires. When the burned area of a wildland fire overlaps with the WUI perimeter, it means that the wildfire has caused damage to a human community, which has been increasing in recent times but still fairly uncommon^9^. The majority of wildfires were still confined to the wildland. Speaking of the risk of human community being effected by wildfires, the number of houses contained within the WUI was also calculated, and the results show that WUI-RS contains the highest proportion of houses, and the housing percentage in WUI-USFS is close to that in WUI-RS. Given that houses in WUI areas are at a high risk of firebrand ignition, these results demonstrate that the WUI-RS can be used to compute the risk from wildland fires near communities.

**Table 2:** Wildfire ignition points, burned area and housing within different WUIs in CA.

	Total area (km$^{2}$)	Number of fires igniting in WUI	Percentage of fires igniting in WUI ($\%$)	Burned area in WUI (km$^{2}$)	Percentage of burned area in WUI ($\%$)	Number of houses in WUI	Percentage of houses in WUI ($\%$)
WUI-RS	28,575	4277	86.25	524.45	4.09	4,959,028	45.13
WUI-USFS (2020)	29,343	4523	66.50	963.29	7.16	4,841,685	43.28
WUI-USFS (2010)	27,255	4414	65.71	951.06	6.70	4,457,884	40.57
WUI-FRAP (2010)	9581	2877	42.83	3.9	0.03	709,198	6.45

Wildfire ignition points from USFS and wildfire perimeters from CAL FIRE were extracted to evaluate the percentage of wildfires ignited or burned within different WUI maps. WUI-RS and WUI-USFS (2020) used wildfire data in 2010–2019, WUI-USFS (2010) and WUI-FRAP(2010) used wildfire data in 2000–2019.

## Conclusion

The development of remote sensing technologies and the wide applications of remote sensing data provides opportunities to improve the accuracy and update frequency of WUI maps. So far, remote sensing data with 30 m resolution has been readily available, with the highest resolution reaching up to 3–5 m, which is enough to clearly delineate the boundary of man-made structures and rendering fine-scale WUI maps. With the ability to obtain high-resolution satellite images, WUI maps are expected to be generated in real time and automatically, which is also the direction of this research in the future.

The new WUI mapping method proposed in this research goes back to the essential definition of WUI, identifies appropriate thresholds for WUI operational definition, and simplifies the requirements of WUI mapping by using modern advanced databases. The vegetation cover of 40$\%$ and the distance of 2.4 km between the wildland vegetation and the housing were validated as the threshold for WUI mapping.

In the new WUI map (WUI-RS), the WUI area in the San Diego county is prominent. When it comes to WUI percentage, the counties with the highest percentage of WUI are concentrated in northern California, west of the Sierra Nevada Mountains. Compared to the two previous WUI maps from USFS, the new WUI map overlaps with them, but with the addition of low density housing clusters surrounded by high-density vegetation, which have been shown to be at a higher wildfire risk than areas with high-density houses. Besides, the WUI-RS captures the highest percentage of ignition points of human-caused wildfires and highest percentage of housing among the three maps, demonstrating its capability to be used for wildfire risk calculation. Through the detailed fire risk analysis within WUI areas, we can conclude that the WUI map generated using our proposed method have the ability to capture the area with high fire risks.

The innovation of this approach is mainly reflected in three aspects: (1) Using remote sensing data to map WUI removes the reliance on census data which is only available every ten years and cannot be updated frequently to keep up with the pace of rapid WUI proliferation. (2) The ability to map individual household footprints provides us the ability to map WUI much more precisely and changes the paradigm from previous WUI mapping approaches which could only locate zones based on housing density as opposed to individual buildings. Using our approach, local authorities will be able to identify wildfire risk down to the level of individual streets and buildings. Although the use of Microsoft building footprint data in WUI mapping has been proposed by other studies^27^, their approach only extracted building locations to calculate the housing density. Moreover, they still followed the existing WUI mapping method in the US. In contrast, we used building footprint data in WUI mapping directly allowing for more precise mapping. (3) We developed the threshold criteria for mapping WUI based on statistical analysis (the OFAT approach) to accommodate the new type of data as opposed to using ad-hoc criteria as used in previous mapping approaches. In this way, WUI maps can incorporate the presence of man made-structures within or at the vicinity of high density vegetation areas that are characterized by high risks of igniting fires regardless of the density of structures. Mapping the WUI areas directly using the housing footprints makes both the new method and the new map intuitive and easier to interpret. Removing the housing calculation not only simplifies the WUI mapping process significantly, but also gets rid of the zoning issue that both the zonal-based and the point-based mapping methods would be troubled by. The rapid update frequency of remote sensing data also makes it possible to update WUI maps frequently without having to rely on the availability of census and zoning data. In terms of the wildland vegetation identification, we collected the fuel vegetation cover from the LANDFIRE database, which provides the vegetation cover in percentage directly based on the satellite image using deep learning algorithm. Adopting this dataset we remove the requirement for vegetation density calculation and do not need to check the land use classifications or the ownership of lands.

While the new method greatly simplifies the calculation of WUI, especially eliminating the need to calculate housing density and vegetation cover, we did not calibrate the vegetation cover data from LANDFIRE. In addition, the new threshold criteria for housing density and vegetation cover developed in this study is only applicable to California, and it needs to be specifically calibrated if mapping in other regions. Therefore, the new method is suitable for mapping high-precision WUI maps to assist in developing targeted management strategies, but is not ideal for large spatial scale (national) WUI mapping and comparison. Meanwhile, the new WUI map cannot be directly compared with the previous WUI maps to show the increase or decrease of WUI area or the change of the spatial pattern.

Considering the application of the WUI map in fire risk visualization, in the future, fire occurrence statistics can also be incorporated into WUI mapping. In this way, fire hazard can be classified more intuitively. Leveraging the rapid development of statistical methods, especially the development of semantic segmentation, using machine learning techniques to streamline and automate the WUI identification process would be a promising future direction. It will reduce computational cost significantly and improve the updating efficiency of WUI maps. Besides, although human activities have become one of the main causes of wildfires, wildfire risk is not a simple combination of human activities and flammable fuels. In our future research, we will combine multiple data layers such as wildfire risk, human activities and flammable fuels, coupled with the WUI mapping approach developed in this work, which will be easier to apply and generalize on a national scale. In addition, WUI maps could have other applications beyond determining wildfire risk, such as analyzing de-urbanization trends or observing the proliferation of invasive plants. For WUI maps with different application purposes, corresponding data or layers can be added in the mapping process to enhance its pertinence.

## Methods

### Housing footprint data

**Figure 6:**
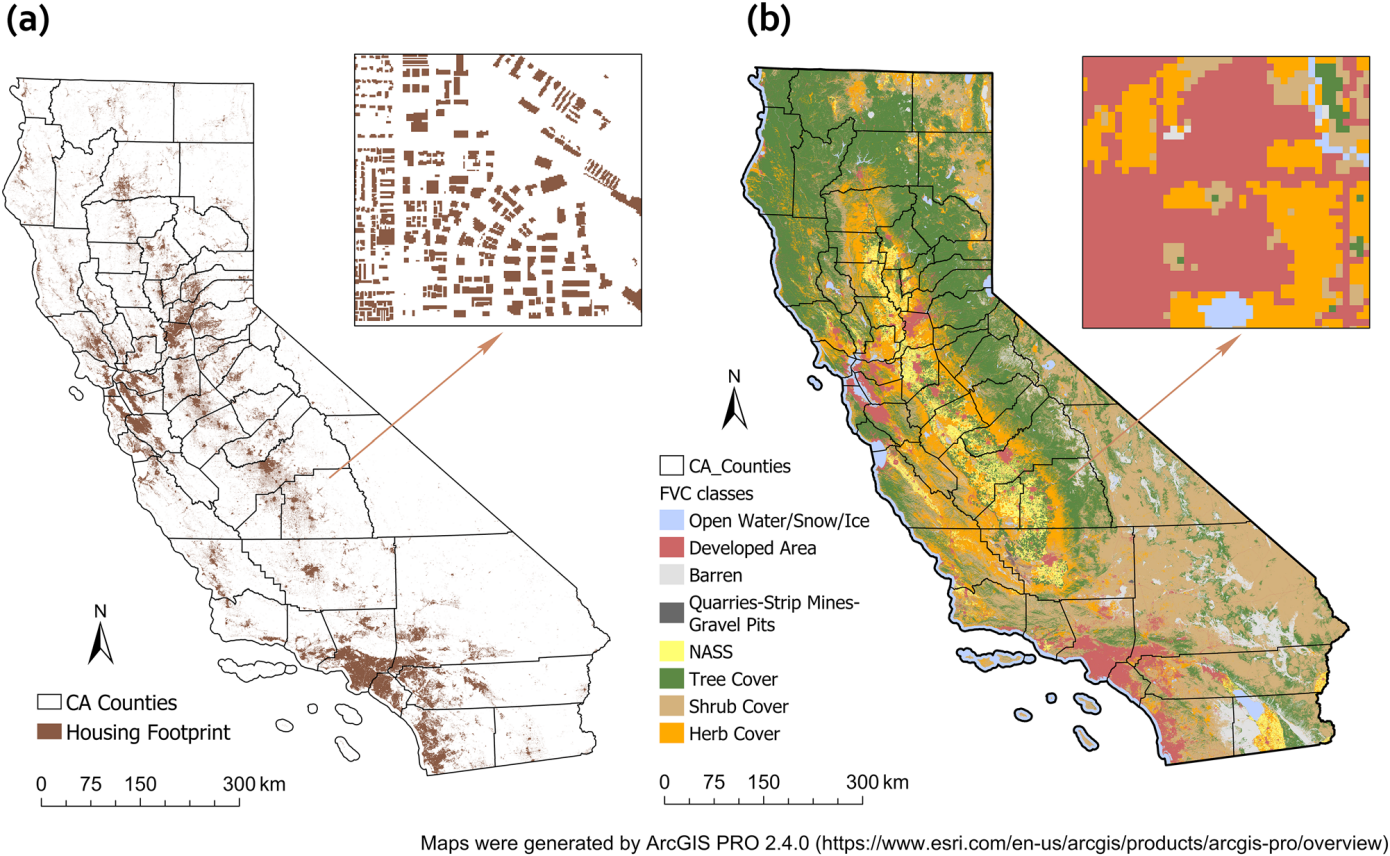
Housing footprint and fuel vegetation cover data in California: (**a**) Housing footprint distribution from Microsoft United States (US) building footprints database, the inset shows the detailed housing layout and shape; (**b**) fuel vegetation cover distribution from LANDFIRE database. The dark blue boundary represents the simplified wildland vegetation boundary which was delineated by selecting pixels with vegetation cover percentage of 50$\%$ or higher, the inset shows the detailed distribution of fuel vegetation. Maps were generated by ArcGIS Pro 2.4.0 (https://www.esri.com/en-us/arcgis/products/arcgis-pro/overview).

One of the most important components of WUI is the presence of human population, which was represented using housing footprints in this study. The housing footprint data in GeoJSON format were obtained from Microsoft United States building footprints database released in 2018. It contains 10,988,525 computer generated building footprints in California, which were extracted from satellite imagery by semantic segmentation and converted to polygons by polygonization^24^. Based on this algorithm, aerial data can also be used to identify building footprints in the future. Thus, remote sensing data in this research refers to the aerial and satellite images of the Earth taken from the air and from space. Due to the large size of GeoJSON format data (2537 MB), it was first split into multiple files in Python before converting to shapefiles in the software ArcGIS Pro (hereinafter ArcGIS), then the shapefile fragments were merged into a complete file. To reduce the computation time and cost, the polygons of housing footprints in the shapefile were converted to 5 meter resolution rasters in the mapping process. The distribution of housing footprints in the entire state of California is shown in Fig. 6a, the inset zoom into a random human community, showing the detailed shape and the layout of extracted housing footprint.

### Vegetation data

To simplify the calculation of vegetation density, the fuel vegetation cover in 2018 from the Landscape Fire and Resource Management Planning Tools (LANDFIRE)^28^ were used as the vegetation information. The 30-m resolution fuel vegetation cover layer (FVC) from the LANDFIRE program adopted “plot-level ground-based visual assessments and lidar observations”, providing the information of the canopy cover of herbaceous, shrub and tree in percentages^28^. Simplified high-density wildland vegetation profiles can be delineated by selecting an appropriate threshold of vegetation cover percentage. The percentage from 20 to 80$\%$ with stride of 10$\%$ were tested in this study by sensitivity analysis. Moreover, the land cover information except for the fuel vegetation, such as the developed area, the barren land, the open water and the snow/ice etc., were also included, which were used to identify the non-wildland area. The distributions of the original FVC as well as the wildland and non-wildland areas are shown in Fig. 6b, the inset zoom into the same resolution as the inset in Fig.6b, showing the detailed distribution of fuel vegetation within the same region.

### WUI mapping method

Based on the definition of WUI from the Federal Register^15^ and the operational definitions from the research of Stewart et al.^16^ and the research from Kumar et al.^9^, WUI refers to the area where the houses are adjacent to (interface) or surrounded by (intermix) high-density fuel vegetation cover. Thus, WUI maps in this study delineated the perimeters of the WUI area and show the classification of intermix and interface area.

WUI was determined based on the presence of houses, the percentage of vegetation cover and the distance between houses and high-density vegetation. To determine the threshold of fuel vegetation cover density and the distance from housing footprints to high-density vegetation, the one factor at a time (OFAT) method was adopted to evaluate the effect of their variations on the total WUI area and the percentage of the wildfire ignitions and burned area contained in the corresponding WUI to the total ignitions and burned area^5,16^. The vegetation cover percentage were tested from greater than 10$\%$ to greater than 90 $\%$ with stride of 10$\%$. In terms of the distance from vegetation to housing footprint, conceptually, the distance of 2.4 km (1.5 mile) is appropriate because it represents statistically how far the firebrands can fly from the fire front and was evaluated and used in several previous studies^5,29^. To prove that the 2.4 km is still applicable in this method, the distance of 1.2 km and 4.8 km were also tested to show the variation in the resultant WUI.

**Figure 7:**
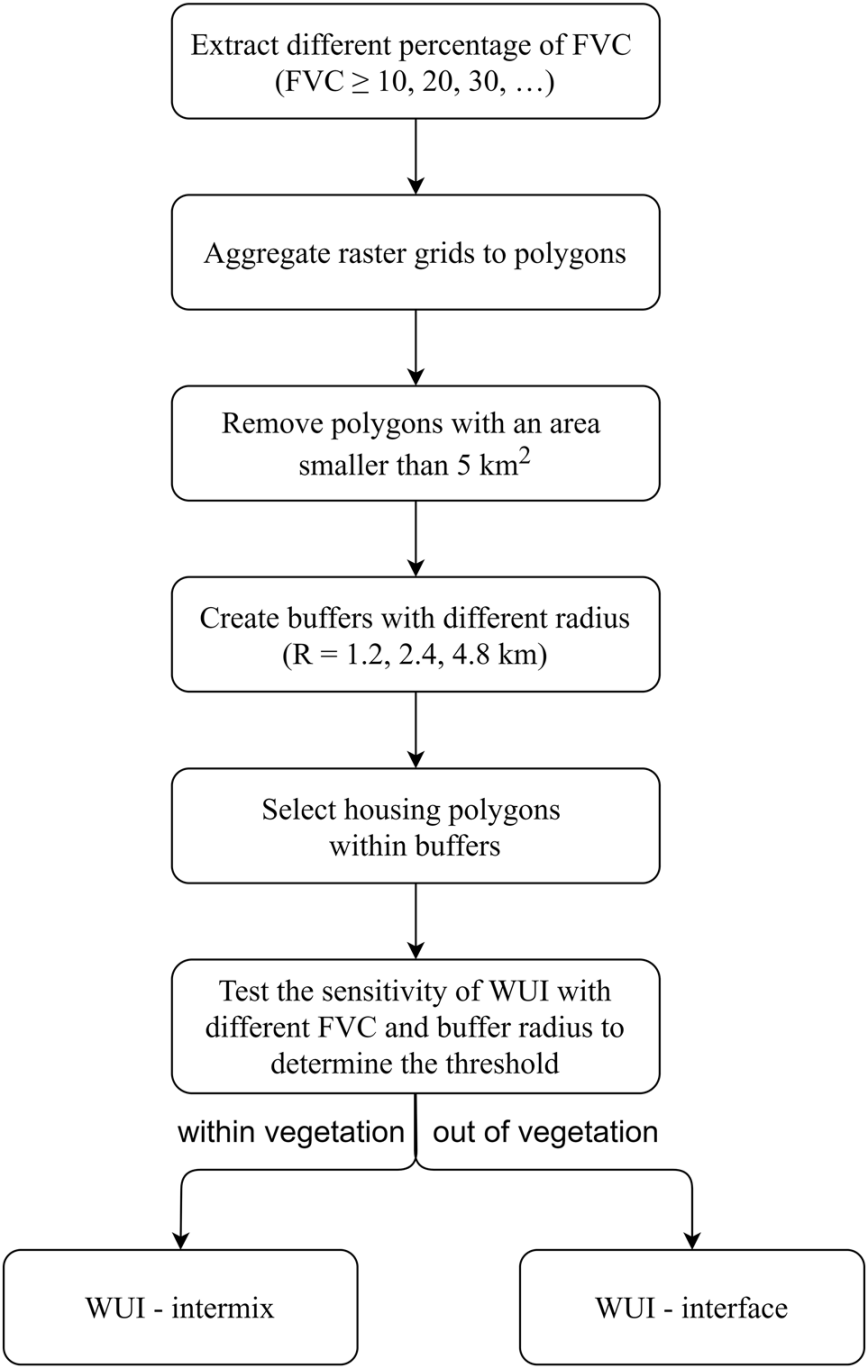
Flowchart of WUI-RS mapping method. FVC represents the fuel vegetation cover and WUI represents the wildland urban interface.

When mapping the WUI, the high-resolution distribution of fuel vegetation cover was extracted from the LANDFIRE dataset based on the selected threshold at the beginning. To exclude the parks, recreational green spaces and green belts in the urban area, the vegetation area with a continuous area of less than 5 $\mathrm {km^{2}}$ were removed. Subsequently, buffers with 2.4 km (or 1.2 km, 4.8 km) radius were created around the vegetation profile. Selecting all the houses located in the high-density vegetation cover (intermix) and buffer zone (interface), the new WUI map was obtained after aggregating the dispersed houses to a continuous area, and it is referred to as WUI-Remote Sensing (abbreviated as WUI-RS). The flowchart of mapping process is shown in Fig. 7.

The new WUI map (WUI-RS) generated in this study was compared to the WUI data products from 2010 (when the last Census data were available) developed by the United States Forest Service (WUI-USFS (2010)) and the California Department of Forestry and Fire Protection’s Fire and Resource Assessment Program (WUI-FRAP (2010)). Using Census data in 2020, we also updated the traditional WUI map following the mapping method used by USFS (WUI-USFS (2020)). The last three maps used threshold of housing density as one of the basic criteria. The criterion in WUI-USFS is 6.17 houses per $\mathrm {km^{2}}$, which is equivalent to one house per 40 acres, whereas the minimum value of housing density within WUI-FRAP is one house per 20 acres. Regarding wildland vegetation, WUI-USFS calculated the percentage of vegetation in each Census block using NLCD data, while WUI-FRAP used the layer of vegetation cover and fire hazard zone from CAL FIRE to determine whether the area is dominated by vegetation. However, the WUI-FRAP added a new classification of “Influence zone”, which involved the vegetation within 1.5 miles (2.4 km) from the interface and the intermix. In general, the criteria for WUI-FRAP are more stringent than that for WUI-USFS. Consequently the total WUI area in California in the WUI-USFS (2010) is 27,255 $\mathrm {km^{2}}$ while in the WUI-FRAP (2010) is 9581 $\mathrm {km^{2}}$. And the area including the influence zone in the WUI-FRAP (2010) is 71,609.22 $\mathrm {km^{2}}$.

### Geospatial analysis methods

To show the spatial distribution of WUI area in each county in California and provide basic statistics information, we split the WUI area along California county boundary. WUI areas in each county were counted and the percentage of WUI areas in each county were calculated by dividing WUI areas by the county area.

To show the consistency and inconsistency between the WUI-RS and WUI-USFS (2020), the overlaps between these two maps were extracted. Also the proportion of overlap areas in the two maps were calculated by dividing the overlap areas by total WUI areas.

**Figure 8:**
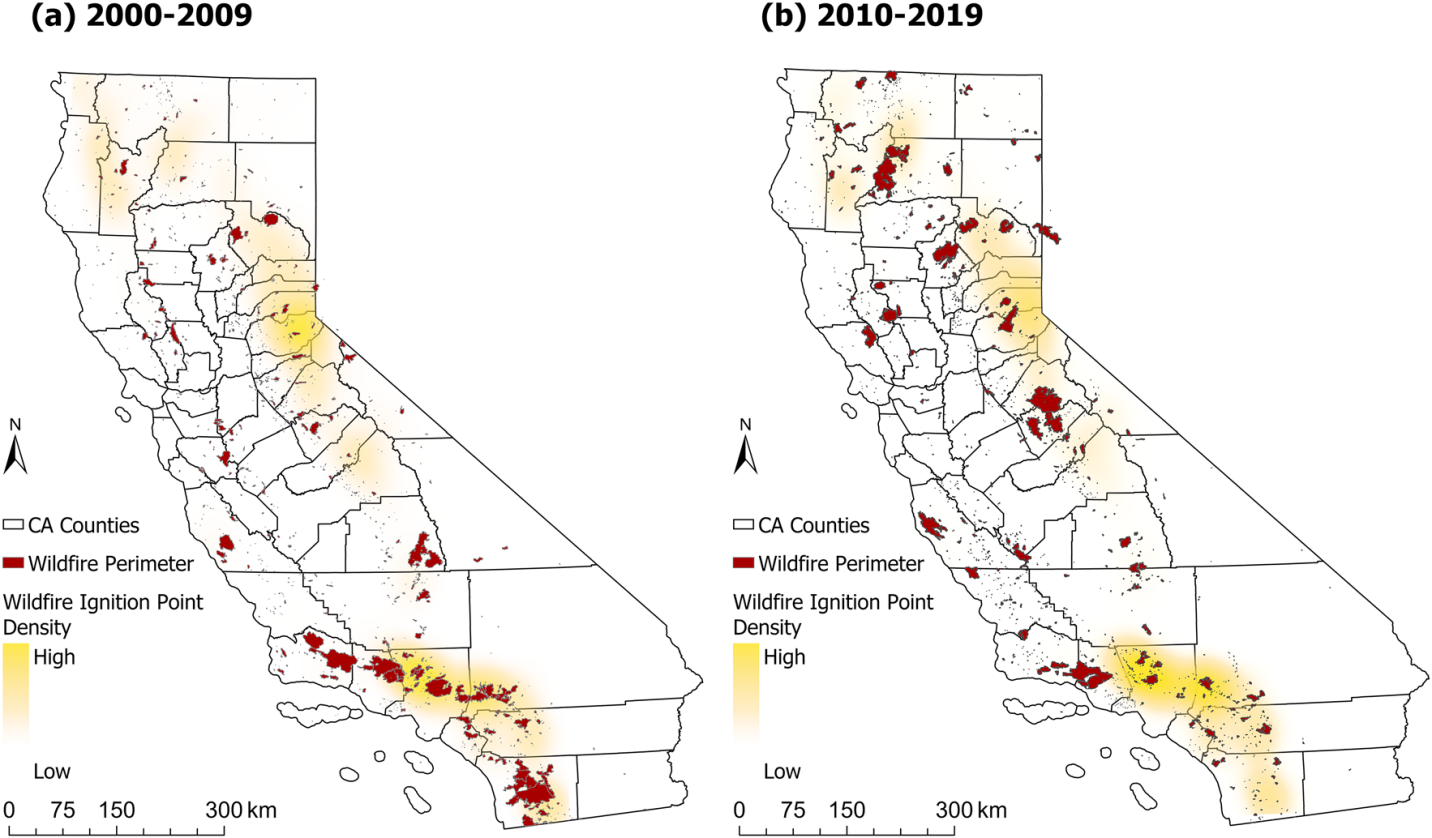
Human-caused wildfire ignition points and perimeters between (**a**) 2000–2009 and (**b**) 2010–2019 in California.

To evaluate the fire risk and its trend in WUI areas, the wildfire ignition points from USFS^30^ and the wildfire perimeters from CAL FIRE^31^ in California were plotted in the maps. The fire ignition point database from USFS dates back to 1970. It includes the ignition points of individual wildfires under the jurisdiction of USFS. The burned area of individual fires varies from smaller than 0.01–410,203 acres (1660 $\mathrm {km^{2}}$). The fire perimeter database from CAL FIRE collected large wildfires under the jurisdiction of CAL FIRE and USFS from 1950. The minimum burned area of the collected wildfires is 10 acres. Since the WUI area has grown rapidly in the last decade since 1990^11^, the 2000–2009 wildfires were used to calculate the percentage of wildfires within the WUI area for the 2010 WUI maps (WUI-USFS and WUI-FRAP), while the new WUI map (WUI-RS) used the 2010–2019 wildfires. The spatial distributions of wildfire ignition points and perimeters between 2000–2009 and 2010–2019 are shown in Fig. 8.
